# miR-486 Promotes Capan-2 Pancreatic Cancer Cell Proliferation by Targeting Phosphatase and Tensin Homolog Deleted on Chromosome 10 (PTEN)

**DOI:** 10.3389/fgene.2019.00541

**Published:** 2019-06-14

**Authors:** Lu Xia, Meiyi Song, Mengxue Sun, Wei Chen, Changqing Yang

**Affiliations:** ^1^ Division of Gastroenterology and Hepatology, Digestive Disease Institute, Shanghai Tongji Hospital, Tongji University School of Medicine, Shanghai, China; ^2^ Emergency Department, Shanghai Tongji Hospital, Tongji University School of Medicine, Shanghai, China

**Keywords:** miRNAs, pancreatic cancer, PTEN, proliferation, therapy

## Abstract

**Introduction:** Pancreatic cancer is one of the most common malignant digestive system tumors. Current treatment options for pancreatic cancer cannot achieve the expected curative effect. MicroRNAs (miRNAs and miRs) participate in many biological and pathological processes. miR-486 has been reported to be involved in diverse types of malignant tumors; however, its role in pancreatic cancer remains unclear.

**Material and Methods:** miR-486 mimics and inhibitors were transfected into Capan-2 cells to increase or decrease the expression of miR-486. Western blot was used to detect protein expression levels. EdU proliferation assay and flow cytometry were applied to identify changes in proliferation. In combination with a PTEN overexpression plasmid, miR-486 mimics were used to determine whether PTEN upregulation abolished the proliferative effect of miR-486.

**Results:** Overexpression of miR-486 promoted proliferation and cell cycle progression of Capan-2 cells. Conversely, the proliferation and cell cycle of Capan-2 cells were attenuated after inhibition of miR-486. Using a combination of bioinformatics and Western blot analysis, PTEN was identified as a downstream target gene of miR-486. The effect of miR-486 on Capan-2 cell proliferation could be abolished by PTEN overexpression.

**Conclusions:** miR-486 promotes the proliferation of Capan-2 cells by targeting PTEN. Inhibition of miR-486 might be a novel therapy for pancreatic cancer.

## Introduction

Pancreatic cancer is a leading cause of mortality worldwide, with characteristic insidious onset, rapid progression, and low survival rate. The mortality rate of pancreatic cancer ranks fourth among all malignant tumors in the US (Jemal et al., 2003; Siegel et al., 2017, 2018) and is predicted to rank second in Western countries by the year 2030 (Rahib et al., 2014; Kuroczycki-Saniutycz et al., 2017). Surgical resection is the only possible treatment to improve long-term survival rate of patients with pancreatic cancer at present (Ryan et al., 2014; Attiyeh et al., 2016). Thus, novel therapies are desperately needed.

MicroRNAs (miRNAs, miRs) are a class of single-stranded non-coding RNAs that play a regulatory role in different tissues/organs and in different growth stages. They participate in many biological and pathological progresses, including embryo development, cell proliferation and apoptosis, and metabolism (Lee et al., 1993; Ambros, 2004; Lewis et al., 2005; Ksiazek-Winiarek et al., 2013; Eaton et al., 2018; Wang et al., 2018). In addition, multiple miRNAs have been reported to play major roles in the development of tumors. miR-486 has been found to be involved in many malignant tumors such as gastric cancer, lung cancer, liver cancer, cervical cancer, and ovarian cancer (Hang et al., 2009; Braconi et al., 2010; Yujuan et al., 2010; Hue-Kian et al., 2011; Res, 2011; Yong et al., 2013; Parikh et al., 2014; Guo et al., 2015; Hou et al., 2016; Roh and So, 2017; Guan et al., 2018), nevertheless, its role in pancreatic cancer is poorly understood.

Here, we found that miR-486 overexpression promoted the proliferation of human pancreatic cancer cell line Capan-2, while miR-486 inhibitors attenuated proliferation. Phosphatase and tensin homolog deleted on chromosome 10 (PTEN) was identified as a target gene of miR-486 in Capan-2 cells, mediating its pro-proliferation effects. Our results suggest that inhibition of miR-486 might be a novel therapy for pancreatic cancer.

## Materials and Methods

### Cell Culture

Human pancreatic cancer cell line Capan-2 was acquired from the Shanghai Cell Bank of Chinese Academy of Sciences (China). Capan-2 cell was cultured with RPMI-1640 medium (keyGEN, Jiangsu, China) containing 10% fetal bovine serum (Gibco, USA) and 1% penicillin-streptomtcin solution (Solarbio, Beijing). The cell was cultured at 37°C in the incubator containing 5% CO_2_ and the morphology was observed by microscope.

### miRNA Transfection

Capan-2 cells were transfected with miR-486 mimics (50 nM, RiboBio, Guangzhou, China), miR-486 inhibitors (100 nM, RiboBio, Guangzhou, China), PTEN overexpression plasmid, or the respective negative controls after 6 h of starvation using Lipo2000 (Invitrogen, Carlsbad, CA, USA). Cells were harvested at 48 h after transfection. The cloning primer sequences for PTEN were designed as follows: has-PTEN-F: CGC GGA TCC ATG TTA GAA GTG GTG ACC TCAC; has-PTEN-R: CCG GAA TTC CTA TAG ATA CTC CAG TTC CAGG.

### Western Blot

Cells were lysed in RIPA buffer supplied with 1 mM PMSF (Invitrogen, Carlsbad, CA, USA) to obtain cell protein. After 30 min on ice, the supernatant was collected by centrifugation at 12,000 rpm for 15 min. Protein concentration was determined using a BCA assay kit (Takara, Japan), and equivalent amounts of protein were resolved by SDS-PAGE and transferred to PVDF membranes (Roche, USA) using a wet transfer method. Membranes were blocked with 5% (w/v) skim milk powder for 2 h and subsequently incubated with primary antibody overnight at 4°C. Primary antibodies used in this study were PTEN (ABclonal, USA, 1:1,000), and GAPDH (Bioworld, USA, 1:1,000). Membranes were next incubated with appropriate secondary antibody (Jackson, USA, 1:10,000) at 25°C for 2 h. ECL detection reagent (Tanon, Shanghai, China) and a fully automated chemiluminescence image analysis system (Tanon, Shanghai, China) were used to visualize the signals. Image J (National Institutes of Health) was used for gray value measurement and statistics.

### Cell Proliferation Assay

Capan-2 cells were incubated with 50 μM 5-ethynyl-2′-deoxyuridine (EdU) for 2 h, and then fixed in 4% (v/v) paraformaldehyde for 30 min. Cells were treated with 2 mg/ml glycine solution and washed with PBS thoroughly to remove paraformaldehyde. After permeabilization, cells were incubated with Apollo dye solution (keyGEN, Jiangsu, China) for 30 min and all nuclei were counterstained with hoechst33342 (keyGEN, Jiangsu, China) for 20 min. An inverted fluorescence microscope (Leica, Germany) was used for obtain florescent images and Image J was used to calculate the ratio of EdU positive cells to total nuclei.

### Analysis of Cell Cycle by Flow Cytometry

At the end of experiment, cells suspensions were prepared by trypsinizing for 1–2 min, which were subsequently mixed with absolute ethyl alcohol and fixed at −20°C overnight. The fixed cells were incubated with a 100 μg/ml propidium iodide (Sigma, USA, P4170) solution supplied with 100 μg/ml RNase A (keyGEN, Jiangsu, China). Cell distribution at different fluorescence intensities was analyzed using a Beckman flow cytometer. FlowJo 7.0 was used to calculate the percentage of cells in G1/G0, S, and G2 phase.

### Real-Time Quantitative Polymerase Chain Reaction

Total RNA of cells was extracted by Total RNA Extractor (Takara, Japan). Extracted RNA was dissolved in RNase-free water and the concentration was measured using a NanoDrop spectrophotometer. To quantify the expression of miR-486 in cells, cDNA was first synthesized by cDNA synthesis kit (Bio-rad, USA). Then SYBR Green reagent (Bio-Rad, USA) and Bulge-LoopTM miRNA qPCR primers (RiboBio, Guangzhou, China) were used together to determine the expression levels of miR-486 on a Bio-Rad CFX-96 Touch Real-Time PCR Detection System. The expression level of miR-486 was normalized to U6 and quantified by the 2^−ΔΔCT^ method. The sequences were as follow: U6 forward: 5-CTCGCTTCGGCAGCACA-3; U6 reverse: 5-AACGCTTCACGAATTTGCGT-3; miR-486 forward: 5-ACACTCCAGCTGGGTC CTGTACTGAGCTGCCC-3; miR-486 reverse: 5-CTCAACTGGTGTCG TGGAGTC GGCAATTCAGTTGAGCCCCGAG-3.

### Statistical Analysis

All data were repeated for three times and presented as means ± SEM. The difference between two groups was compared by two-tailed unpaired student’s t test, and a significance test of mean difference between two or more samples was conducted by one-way ANOVA followed by Bonferroni test. Differences were considered to be statistically significant when the *p* was <0.05.

## Results

### miR-486 Promotes Cell Proliferation and Cell Cycle Progression in Capan-2 Cells

**Figure 1:**
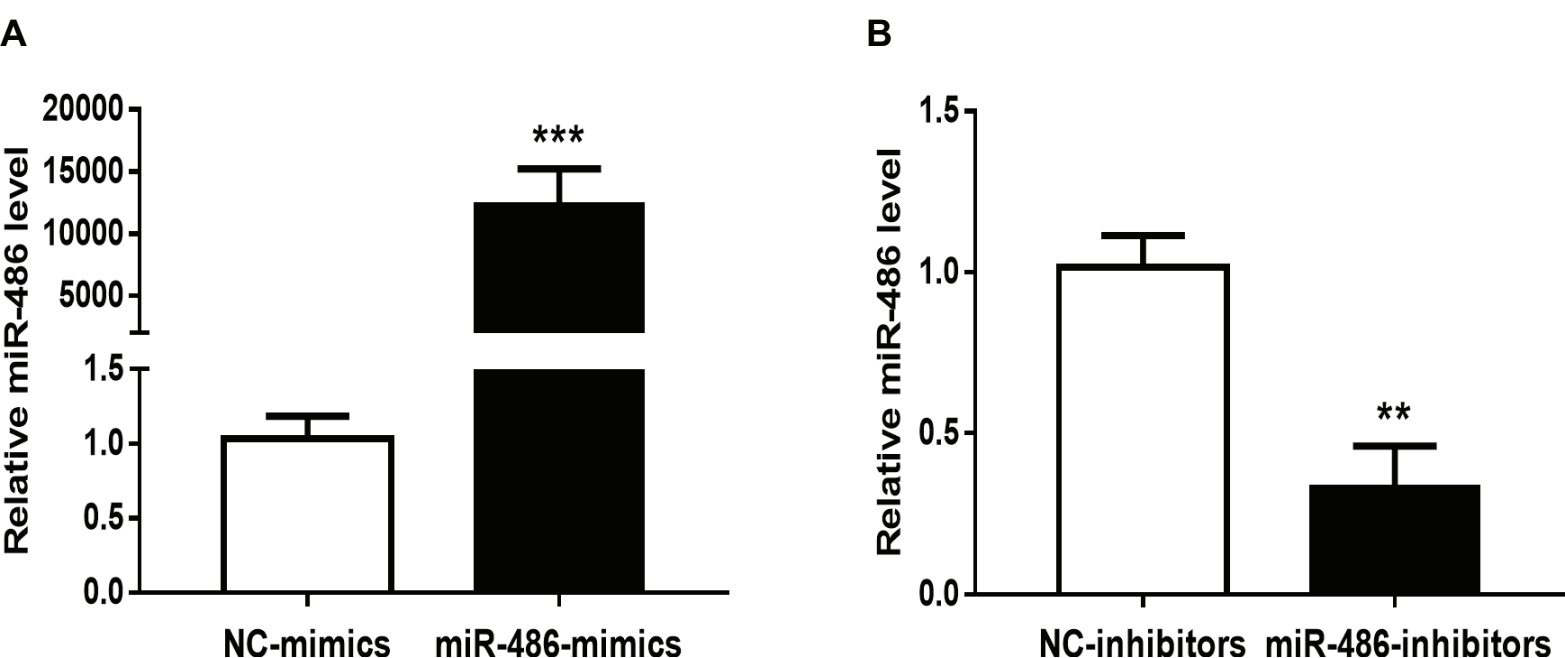
Effects of miR-486 mimics and inhibitors on miR-486 expression in Capan-2 cells. **(A)** miR-486 mimics significantly increase the expression of miR-486 in Capan-2 cells. **(B)** miR-486 inhibitors significantly decrease the expression of miR-486 in Capan-2 cells. **p < 0.01; ***p < 0.001. *n* = 4 per group.

**Figure 2:**
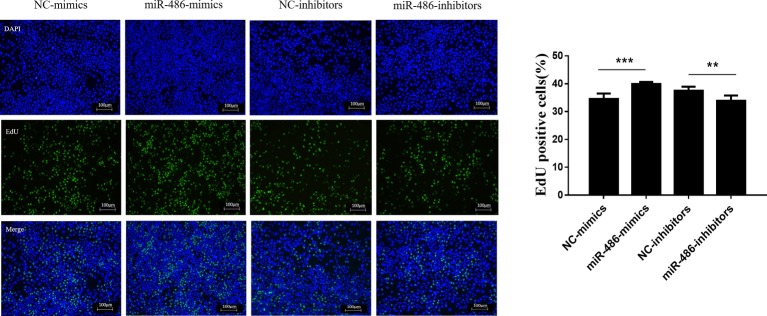
miR-486 promotes the proliferation of Capan-2 cells. EdU proliferation assay shows that miR-486 mimics promote the proliferation of Capan-2 cells, while miR-486 inhibitors inhibit that. Scale bar: 100 μm. **p < 0.01; ***p < 0.001; *n* = 6 per group.

**Figure 3:**
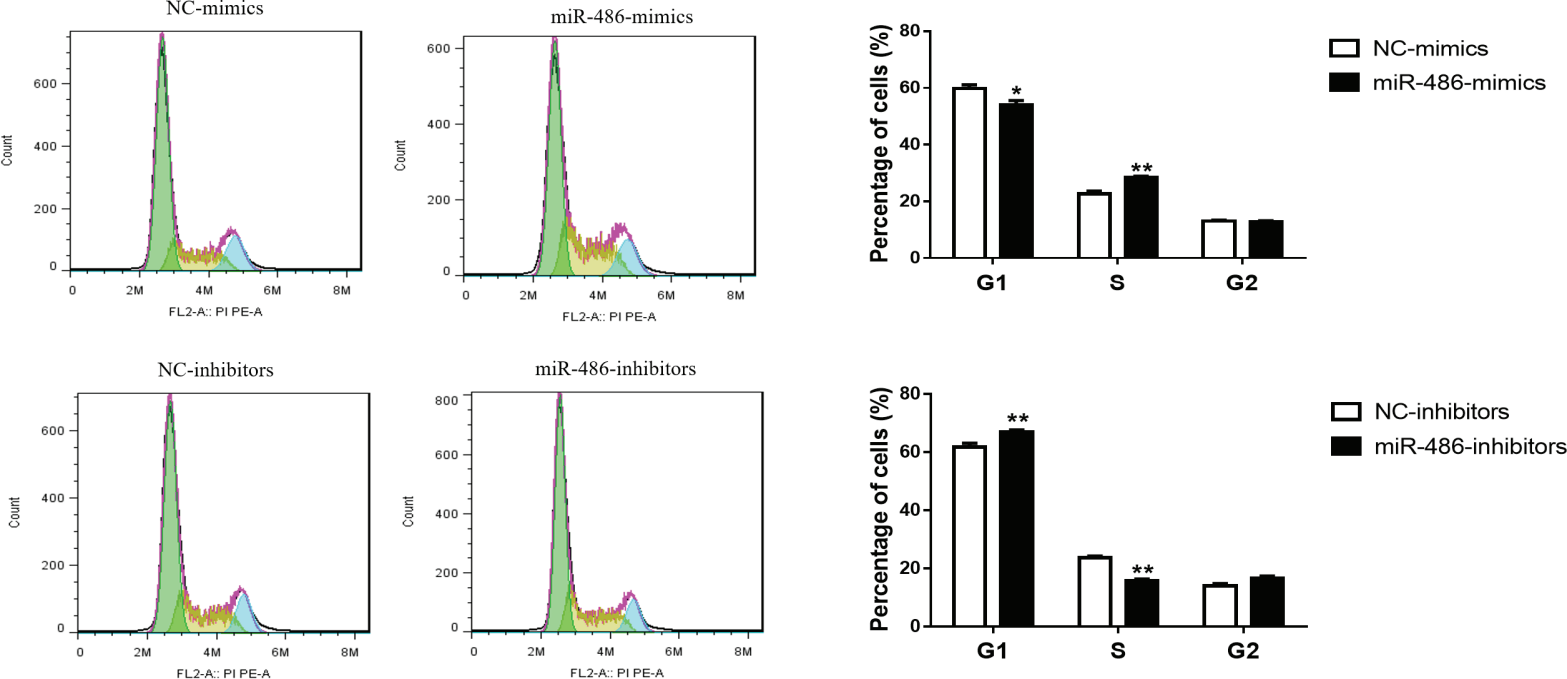
miR-486 promotes the cell cycle progression of Capan-2 cells. PI cell cycle assay shows that miR-486 mimics promote the progression of Capan-2 cells from G1 to S phase while miR-486 inhibitors inhibit it. *p < 0.05; **p < 0.01; *n* = 6 per group.

To investigate the role of miR-486 in Capan-2 cell proliferation, miR-486 mimics and inhibitors were used. Real time quantitative PCR confirmed that miR-486 mimics significantly increased the expression of miR-486 (Figure 1A), while miR-486 inhibitors decreased its expression (Figure 1B). As determined by EdU staining, miR-486 mimics significantly increased the number of EdU positive cells, while miR-486 inhibitors decreased the number of EdU positive cells, indicating that miR-486 mimics could promote the proliferation of Capan-2 cells and miR-486 inhibitors could block it (Figure 2). Moreover, flow cytometry revealed that miR-486 mimics promoted cell progression from G1 to S phase, whereas miR-486 inhibitors decreased it (Figure 3). Collectively, these data suggested that miR-486 promoted cell proliferation and cell cycle progression in Capan-2 cells.

### PTEN Is a Target Gene of miR-486 in Capan-2 Cells

**Figure 4:**
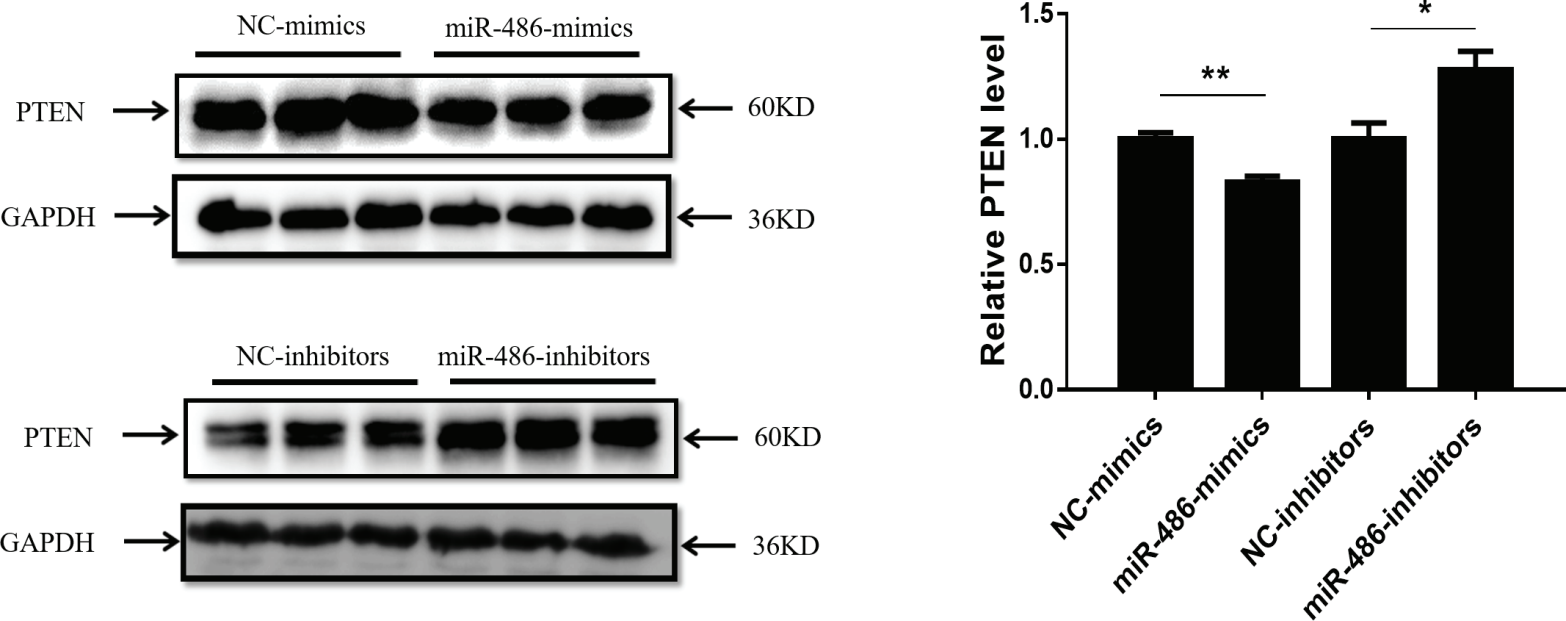
PTEN is negatively regulated by miR-486 in Capan-2 cells. Western blot shows that miR-486 mimics downregulate the expression of PTEN in Capan-2 cells while miR-486 inhibitors upregulate it (Relative to GAPDH). *p < 0.05; **p < 0.01; *n* = 3 per group.

PTEN has been reported to be a target gene of miR-486 in other types of cells (Small et al., 2010; Gao et al., 2018), however, whether PTEN is a target gene of miR-486 in Capan-2 cells has not been confirmed. Capan-2 cells were transfected with miR-486 mimics and inhibitors, and Western blot was used to determine the expression of PTEN at the protein level. It was found that overexpression of miR-486 in Capan-2 decreased the expression of PTEN, while inhibition of miR-486 increased its expression, confirming that miR-486 could regulate PTEN at least at the protein level (Figure 4; Supplementary Figures 1–4).

### Overexpression of PTEN Reverses the Pro-proliferation Effect of miR-486 in Capan-2 Cells

**Figure 5:**
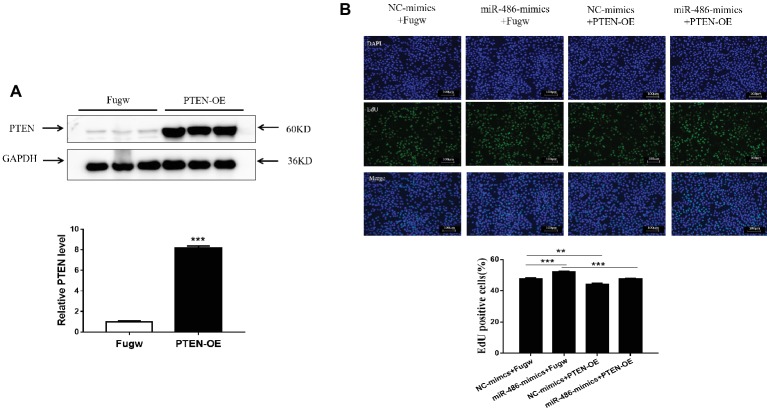
Down-regulation of PTEN mediates the pro-proliferation effects of miR-486 in Capan-2 cells. **(A)** Western blot confirmed that PTEN overexpression (PTEN-OE) plasmid significantly increases PTEN in Capan-2 cells. **, *p* < 0.01. *n* = 3 per group. **(B)** PTEN overexpression reverse the pro-proliferation effect of miR-486 in Capan-2 cells. Scale bar: 100 μm. **p < 0.01; ***p < 0.001; *n* = 6 per group.

To determine whether PTEN influences the proliferation effects of miR-486 in Capan-2 cells, a PTEN overexpression plasmid was used to perform a functional rescue experiment in the presence of the miR-486 mimics (Figure 5A; Supplementary Figures 5, 6). It was found that overexpression of PTEN reduced the number of EdU positive cells. Compared with the group transfected with miR-486 mimics, the number of EdU positive cells in the group transfected with PTEN overexpression plasmid and miR-486 mimics decreased significantly, indicating that overexpression of PTEN could reduce the pro-proliferation effects of miR-486 in Capan-2 cells (Figure 5B). This result confirmed that up-regulation of PTEN mediates the pro-proliferation effects of miR-486 in Capan-2 cells.

## Discussion

As a worldwide public health threat, pancreatic cancer has a metastasis rate of 85–95%, which causes approximately 330,000 deaths annually (Siegel et al., 2015). Surgical resection is currently the most effective treatment, but local recurrence and distant metastasis tend to occur after operation and the 5-year survival rate is less than 20% (Oettle et al., 2007). Gemcitabine is the preferred chemotherapy drug for non-surgical treatment of pancreatic cancer. While chemotherapy drugs tend to prolong the survival of patients with advanced stage pancreatic cancer, such drugs have limitations due to their toxicity and adverse reactions (Thota et al., 2014). In the present study, we demonstrated that miR-486 was involved in the regulation of Capan-2 pancreatic cancer cell proliferation by targeting PTEN. Inhibition of miR-486 might be a novel therapeutic strategy for pancreatic cancer.

miR-486 locates to the region sAnk1 on chromosome 8p11, and interestingly, the abnormal changes of genes in this region are closely related to the pathogenesis of various tumors. miR-486 has been reported to inhibit the proliferation and metastasis of lung cancer, but may play a causative role in cervical and prostate cancer (Yong et al., 2013; Ye et al., 2016; Yang et al., 2017). It has been suggested that miR-486 functions as a tumor suppressor in digestive system cancer including colorectal cancer, esophageal and hepatocellular carcinoma and is highly expressed in colorectal cancer (Small et al., 2010; Mosakhani et al., 2012; Youness et al., 2016; Lang and Zhao, 2018). Tumor suppressor PTEN has been reported to be a regulatory target gene of miR-486, and the regulation axis of miR-486/PTEN has been confirmed in non-small cell lung cancer (Gao et al., 2018). MiR-486 is also considered as the biomarker for the diagnosis and prognosis of multiple tumors (Li et al., 2015, 2018; Jiang et al., 2018). Recently, the expression level of circulating miR-486 was found to be increased in the patients with pancreatic cancer and exhibited good diagnostic value for this disease (Xu et al., 2016). However, the role of miR-486 in pancreatic cancer was to this point less clear. Here, we found that miR-486 overexpression promoted the proliferation of human pancreatic cancer Capan-2 cell line while miR-486 inhibition attenuated such proliferation as determined by EdU staining and flow cytometry cell cycle analysis.

PTEN was the first tumor suppressor gene found to have phosphatase activity. Located on human chromosome 10q23, it can promote the dephosphorylation of the second messenger PIP3, and functions in maintaining cell structure and signal transduction. PTEN is necessary for the body to maintain normal biological processes and to exert anti-cancer effects. A variety of tumors including glioblastoma, prostate cancer, breast cancer, lung cancer, melanoma, and digestive system cancer are associated with the inactivation of PTEN (Furnari et al., 1997; Myers et al., 1997; Maehama and Dixon, 1998; Zhang et al., 2004). PTEN was reported to be down-regulated in pancreatic cancer cell lines and targets NF-kappaB and c-Myc by activating PI3K/Akt signaling pathway, thereby exerting a role in inhibiting pancreatic cancer (Asano et al., 2004; Ying et al., 2011). In addition, PTEN is involved in the regulation of angiogenesis in pancreatic cancer cells and may be related to the chemoresistance and cancer stemness (Gu et al., 2016; Wang et al., 2016). PTEN is a well-documented target of miR-486 in other types of cells (Small et al., 2010; Alexander et al., 2014; Gao et al., 2018); however, due to cell specificity, whether PTEN is a target gene of miR-486 in pancreatic cancer was unclear. In this study, we found that miR-486 could negatively regulate the expression of PTEN at least at the protein level. Importantly, overexpression of PTEN abolished the pro-proliferation effects of miR-486 mimics, suggesting that PTEN is a functional target gene of miR-486 in Capan-2 cells.

In conclusion, miR-486 promotes human pancreatic cancer cell line Capan-2 cell proliferation by targeting PTEN. Therefore, targeting miR-486/PTEN is a potential new therapeutic strategy for pancreatic cancer.

## Data Availability

The raw data supporting the conclusions of this manuscript will be made available by the authors, without undue reservation, to any qualified researcher.

## Author Contributions

LX performed the experiments and wrote the manuscript. MSo and MSu performed the experiments and analyzed the data. WC revised the manuscript. CY designed the study. All authors have read and approved the final manuscript.

### Conflict of Interest Statement

The authors declare that the research was conducted in the absence of any commercial or financial relationships that could be construed as a potential conflict of interest.

## References

[ref1] Alexander M. S. Casar J. C. Motohashi N. Vieira N. M. Eisenberg I. Marshall J. L.. (2014). MicroRNA-486 dependent modulation of DOCK3/PTEN/AKT signaling pathways improves muscular dystrophy-associated symptoms. J. Clin. Investig. 124, 2651–2667. 10.1172/JCI73579, PMID: 24789910 PMC4038577

[ref2] Ambros V. (2004). The functions of animal microRNAs. Nature 431, 350–355. 10.1038/nature02871, PMID: 15372042

[ref3] Asano T. Yao Y. J. Li D. Abbruzzese J. L. Reddy S. A. (2004). The PI 3-kinase/Akt signaling pathway is activated due to aberrant Pten expression and targets transcription factors NF-kappaB and c-Myc in pancreatic cancer cells. Oncogene 23, 8571–8580. 10.1038/sj.onc.1207902, PMID: 15467756

[ref4] Attiyeh M. A. Fernández-Del C. C. Al E. M. Eaton A. A. Gönen M. Batts R. (2016). Development and validation of a multi-institutional preoperative nomogram for predicting grade of dysplasia in intraductal papillary mucinous neoplasms (IPMNs) of the pancreas: a report from the Pancreatic Surgery Consortium. Ann. Surg. 1, 157–163. 10.1097/SLA.0000000000002015 PMC5565720 28079542

[ref5] Braconi C. Huang N. Patel T. (2010). MicroRNA dependent regulation of DNMT-1 and tumor suppressor gene expression by Interleukin-6 in human malignant cholangiocytes. Hepatology 51, 881–890. 10.1002/hep.23381, PMID: 20146264 PMC3902044

[ref6] Eaton M. Granata C. Barry J. Safdar A. Bishop D. Little J. P. (2018). Impact of a single bout of high-intensity interval exercise and short-term interval training on interleukin-6, FNDC5, and METRNL mRNA expression in human skeletal muscle. J. Sport Health Sci. 7, 191–196. 10.1016/j.jshs.2017.01.003, PMID: 30356443 PMC6180539

[ref7] Furnari F. B. Lin H. Huang H. J. S. Cavenee W. K. (1997). Growth suppression of glioma cells by PTEN requires a functional phosphatase catalytic domain. Proc. Natl. Acad. Sci. U.S.A. 94, 12479–12484. 10.1073/pnas.94.23.12479 9356475 PMC25009

[ref8] Gao Z. J. Yuan W. D. Yuan J. Q. Yuan K. Wang Y. (2018). miR-486-5p functions as an oncogene by targeting PTEN in non-small cell lung cancer. Pathol. Res. Pract. 214, 700–705. 10.1016/j.prp.2018.03.013 29567332

[ref9] Gu J. Wang D. Zhang J. Zhu Y. Li Y. Chen H.. (2016). GFRα2 prompts cell growth and chemoresistance through down-regulating tumor suppressor gene PTEN *via* Mir-17-5p in pancreatic cancer. Cancer Lett. 380, 434–441. 10.1016/j.canlet.2016.06.016, PMID: 27400681

[ref10] Guan Z. Tan J. Gao W. Li X. Yang Y. Li X. (2018). Circular RNA hsa_circ_0016788 regulates hepatocellular carcinoma tumorigenesis through miR-486/CDK4 pathway. J. Cell. Physiol. 234, v500–v508. 10.1002/jcp.26612 29923236

[ref11] Guo Q. Zhang H. Zhang L. He Y. Weng S. Dong Z.. (2015). MicroRNA-21 regulates non-small cell lung cancer cell proliferation by affecting cell apoptosis *via* COX-19. Int. J. Clin. Exp. Med. 8, 8835–8841. PMID: 26309536 PMC4538100

[ref12] Hang S. Jian-Rong Y. Teng X. Jun H. Li X. Yunfei Y. (2009). MicroRNA-101, down-regulated in hepatocellular carcinoma, promotes apoptosis and suppresses tumorigenicity. Cancer Res. 69, 1135–1142. 10.1158/0008-5472.CAN-08-2886 19155302

[ref13] Hou L. Jian C. Zheng Y. Wu C. (2016). Critical role of miR-155/FoxO1/ROS axis in the regulation of non-small cell lung carcinomas. Tumor Biol. 37, 5185–5192. 10.1007/s13277-015-4335-9, PMID: 26548866

[ref14] Hue-Kian O. Angie Lay-Keng T. Kakoli D. Chia-Huey O. Nian-Tao D. Iain Beehuat T. (2011). Genomic loss of miR-486 regulates tumor progression and the OLFM4 antiapoptotic factor in gastric cancer. Clin. Cancer Res. 17, 2657–2667. 10.1158/1078-0432.CCR-10-3152 21415212

[ref15] Jemal A. Siegel R. Ward E. Murray T. Xu J. Thun M. J. (2003). Cancer statistics, 2009. CA Cancer J. Clin. 53, 5–26. 10.3322/canjclin.53.1.5, PMID: 12568441

[ref16] Jiang M. Li X. Quan X. Yang X. Zheng C. Hao X.. (2018). MiR-486 as an effective biomarker in cancer diagnosis and prognosis: a systematic review and meta-analysis. Oncotarget 9, 13948–13958. 10.18632/oncotarget.24189, PMID: 29568407 PMC5862628

[ref17] Ksiazek-Winiarek D. J. Kacperska M. J. Glabinski A. (2013). MicroRNAs as novel regulators of neuroinflammation. Mediat. Inflamm. 2013:172351. 10.1155/2013/172351 PMC3745967 23983402

[ref18] Kuroczycki-Saniutycz S. Grzeszczuk A. Zwierz Z. W. Kołodziejczyk P. Szczesiul J. Zalewska-Szajda B.. (2017). Prevention of pancreatic cancer. Contemp. Oncol. 21, 30–34. 10.5114/wo.2016.63043, PMID: 28435395 PMC5385470

[ref19] Lang B. P. Zhao S. (2018). miR-486 functions as a tumor suppressor in esophageal cancer by targeting CDK4/BCAS2. Oncol. Rep. 39, 71–80. 10.3892/or.2017.6064, PMID: 29115564 PMC5783606

[ref20] Lee R. C. Feinbaum R. L. Ambros V. (1993). The C. elegans heterochronic gene lin-4 encodes small RNAs with antisense complementarity to lin-14. Cell 75, 843–854. 10.1016/0092-8674(93)90529-Y, PMID: 8252621

[ref21] Lewis B. P. Burge C. B. Bartel D. P. (2005). Conserved seed pairing, often flanked by adenosines, indicates that thousands of human genes are microRNA targets. Cell 120, 15–20. 10.1016/j.cell.2004.12.035, PMID: 15652477

[ref22] Li W. Wang Y. Zhang Q. Tang L. Liu X. Dai Y. (2015). MicroRNA-486 as a biomarker for early diagnosis and recurrence of non-small cell lung cancer. PLoS One 11:e0148589. 10.1371/journal.pone.0148589 PMC4523212 26237047

[ref23] Li C. Zheng X. Li W. Bai F. Lyu J. Meng Q. H. (2018). Serum miR-486-5p as a diagnostic marker in cervical cancer: with investigation of potential mechanisms. BMC Cancer 18:61. 10.1186/s12885-017-3753-z 29316891 PMC5759341

[ref24] Maehama T. Dixon J. E. (1998). The tumor suppressor, PTEN/MMAC1, dephosphorylates the lipid second messenger, phosphatidylinositol 3,4,5-trisphosphate. J. Biol. Chem. 273, 13375–13378. 10.1074/jbc.273.22.13375, PMID: 9593664

[ref25] Mosakhani N. Sarhadi V. K. Borze I. Karjalainen-Lindsberg M. L. Sundstrom J. Ristamaki R.. (2012). MicroRNA profiling differentiates colorectal cancer according to KRAS status. Genes Chromosom. Cancer 51, 1–9. 10.1002/gcc.20925, PMID: 21922590

[ref26] Myers M. P. Stolarov J. P. Eng C. Li J. Wang S. I. Wigler M. H. (1997). P-TEN, the tumor suppressor from human chromosome 10q23, is a dual-specificity phosphatase. Proc. Natl. Acad. Sci. U.S.A. 94, 9052–9057. 10.1073/pnas.94.17.9052 9256433 PMC23024

[ref27] Oettle H. Post S. Neuhaus P. Gellert K. Langrehr J. Ridwelski K. (2007). Adjuvant chemotherapy with gemcitabine vs observation in patients undergoing curative-intent resection of pancreatic cancer - A randomized controlled trial. JAMA 297, 267–277. 10.1001/jama.297.3.267 17227978

[ref28] Parikh A. Lee C. Joseph P. Marchini S. Baccarini A. Kolev V. (2014). microRNA-181a has a critical role in ovarian cancer progression through the regulation of the epithelial–mesenchymal transition. Nat. Commun. 5, 149–168. 10.1038/ncomms3977 PMC3896774 24394555

[ref29] Rahib L. Smith B. D. Aizenberg R. Rosenzweig A. B. Fleshman J. M. Matrisian L. M. (2014). Projecting cancer incidence and deaths to 2030: the unexpected burden of thyroid, liver, and pancreas cancers in the United States. Cancer Res. 74, 2913–2921. 10.1158/0008-5472.CAN-14-0155, PMID: 24840647

[ref30] Res C. (2011). Correction: serum microRNA profiles serve as novel biomarkers for HBV infection and diagnosis of HBV-positive hepatocarcinoma. Cancer Res. 71, 9798–9807. 10.1158/0008-5472 21098710

[ref31] Roh H. T. So W. Y. (2017). The effects of aerobic exercise training on oxidant-antioxidant balance, neurotrophic factor levels, and blood-brain barrier function in obese and nonobese men. J. Sport Health Sci. 6, 447–453. 10.1016/j.jshs.2016.07.006, PMID: 30356625 PMC6189263

[ref32] Ryan D. P. Hong T. S. Bardeesy N. (2014). Pancreatic adenocarcinoma. N. Engl. J. Med. 371, 1039–1049. 10.1056/NEJMra1404198, PMID: 25207767

[ref33] Siegel R. L. Miller K. D. Dvm A. J. (2017). Cancer statistics, 2017. CA Cancer J.Clin. 67, 7–30. 10.3322/caac.21387, PMID: 28055103

[ref34] Siegel R. L. Miller K. D. Jemal A. (2015). Cancer statistics, 2015. CA Cancer J. Clin. 65, 5–29. 10.3322/caac.21254 25559415

[ref35] Siegel R. L. Miller K. D. Jemal A. (2018). Cancer statistics, 2018. CA Cancer J. Clin. 60, 277–300. 10.3322/caac.21442 20610543

[ref36] Small E. M. O'Rourke J. R. Moresi V. Sutherland L. B. McAnally J. Gerard R. D.. (2010). Regulation of PI3-kinase/Akt signaling by muscle-enriched microRNA-486. Proc. Natl. Acad. Sci. U.S.A. 107, 4218–4223. 10.1073/pnas.1000300107, PMID: 20142475 PMC2840099

[ref37] Thota R. Pauff J. M. Berlin J. D. (2014). Treatment of metastatic pancreatic adenocarcinoma: a review. Oncology 28, 70–74. 10.3892/ol.2013.1655 24683721

[ref38] Wang L. Lv Y. Li G. Xiao J. (2018). MicroRNAs in heart and circulation during physical exercise. J. Sport Health Sci. 7, 433–441. 10.1016/j.jshs.2018.09.008, PMID: 30450252 PMC6226555

[ref39] Wang M. C. Min J. Tao W. Li J. Jie C. Hui G.. (2016). Polycomb complex protein BMI-1 promotes invasion and metastasis of pancreatic cancer stem cells by activating PI3K/AKT signaling, anex vivo,in vitro, andin vivostudy. Oncotarget 7, 9586–9599. 10.18632/oncotarget.7078, PMID: 26840020 PMC4891062

[ref40] Xu J. W. Cao Z. Liu W. J. You L. Zhou L. Wang C. Y.. (2016). Plasma miRNAs effectively distinguish patients with pancreatic cancer from controls: a multicenter study. Ann. Surg. 263, 1173–1179. 10.1097/SLA.0000000000001345, PMID: 26114496

[ref41] Yang Y. Ji C. W. Guo S. H. Su X. Zhao X. Z. Zhang S. W.. (2017). The miR-486-5p plays a causative role in prostate cancer through negative regulation of multiple tumor suppressor pathways. Oncotarget 8, 72835–72846. 10.18632/oncotarget.20427, PMID: 29069829 PMC5641172

[ref42] Ye H. Yu X. Xia J. Tang X. Tang L. Chen F. (2016). MiR-486-3p targeting ECM1 represses cell proliferation and metastasis in cervical cancer. Biomed. Pharmacother. 80, 109–114. 10.1016/j.biopha.2016.02.019, PMID: 27133046

[ref43] Ying H. Elpek K. G. Vinjamoori A. Zimmerman S. M. Chu G. C. Yan H.. (2011). PTEN is a major tumor suppressor in pancreatic ductal adenocarcinoma and regulates an NF-κB–cytokine network. Cancer Discov. 1, 158–169. 10.1158/2159-8290.CD-11-0031, PMID: 21984975 PMC3186945

[ref44] Yong P. Yuntao D. Charles H. Xiaojuan Y. Kassis E. S. Lunxu L. (2013). Insulin growth factor signaling is regulated by microRNA-486, an underexpressed microRNA in lung cancer. Proc. Natl. Acad. Sci. U.S.A. 110, 15043–15048. 10.1073/pnas.1307107110 23980150 PMC3773758

[ref45] Youness R. A. El-Tayebi H. M. Assal R. A. Hosny K. Esmat G. Abdelaziz A. I. (2016). MicroRNA-486-5p enhances hepatocellular carcinoma tumor suppression through repression of IGF-1R and its downstream mTOR, STAT3 and c-Myc. Oncol. Lett. 12, 2567–2573. 10.3892/ol.2016.4914, PMID: 27698829 PMC5038225

[ref46] Yujuan X. Jian-Hong F. Jing-Ping Y. Jine Y. Ying Z. Wei-Hua J. (2010). Effects of microRNA-29 on apoptosis, tumorigenicity, and prognosis of hepatocellular carcinoma. Hepatology 51, 836–845. 10.1002/hep.23380 20041405

[ref47] Zhang L. Yu Q. He J. Zha X. (2004). Study of the PTEN gene expression and FAK phosphorylation in human hepatocarcinoma tissues and cell lines. Mol. Cell. Biochem. 262, 25–33. 10.1023/B:MCBI.0000038212.78008.7f 15532706

