# Supplementary material for: miR-486 Promotes Capan-2 Pancreatic Cancer Cell Proliferation by Targeting Phosphatase and Tensin Homolog Deleted on Chromosome 10 (PTEN)
Source: Front Genet. 2019 Jun 14;10:541. doi: 10.3389/fgene.2019.00541 (PMC6588128; doi:10.3389/fgene.2019.00541)
Supplement: Supplementary file 1 [file Data_Sheet_1.DOCX]

Supplementary Material

## Supplementary Figures


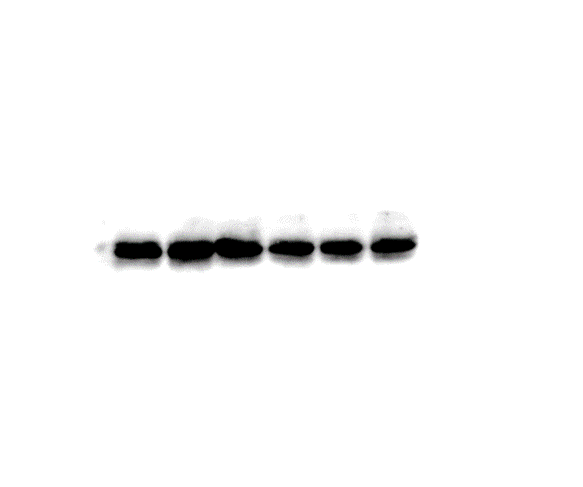


**Supplementary Figure 1.** The expression of PTEN in Capan-2 cells with miR-486 mimic. The three samples on the left are the control group, and the three samples on the right are the miR-486 mimic transfection groups. (blots in Figure 4)


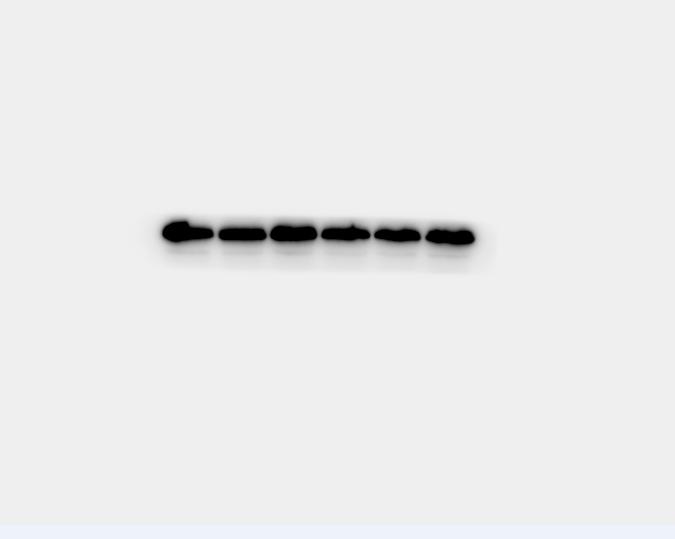


**Supplementary Figure 2.** The expression of GAPDH in Capan-2 cells with miR-486 mimic. The three samples on the left are the control group, and the three samples on the right are the miR-486 mimic transfection groups. (blots in Figure 4)


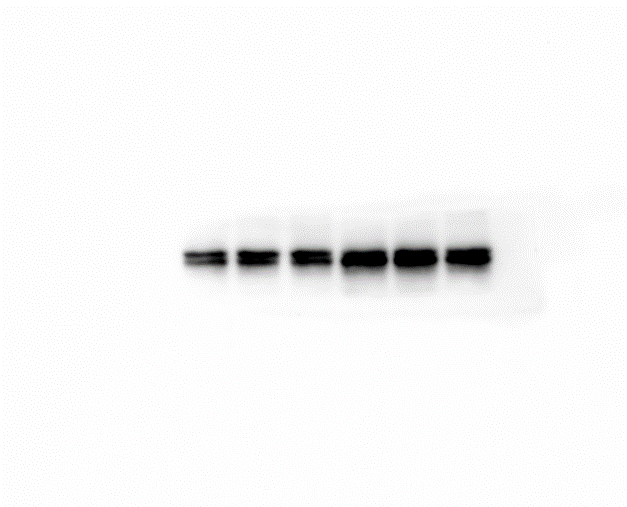


**Supplementary Figure 3.** The expression of PTEN in Capan-2 cells with miR-486 inhibitor. The three samples on the left are the control group, and the three samples on the right are the miR-486 inhibitor transfection groups. (blots in Figure 4)


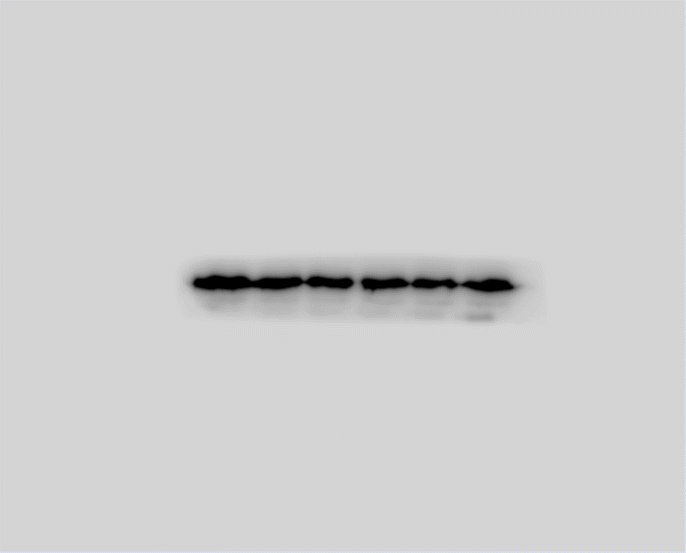


**Supplementary Figure 4.** The expression of GAPDH in Capan-2 cells with miR-486 inhibitor. The three samples on the left are the control group, and the three samples on the right are the miR-486 inhibitor transfection groups. (blots in Figure 4)


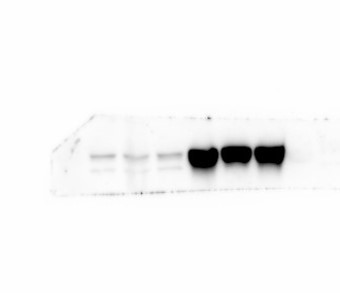


**Supplementary Figure 5.** The expression of PTEN in Capan-2 cells by PTEN overexpression plasmid. The three samples on the left are the control group, and the three samples on the right are the PTEN overexpression plasmid transfection groups. (blots in Figure 5A)


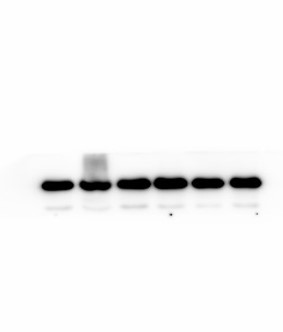


**Supplementary Figure 6.** The expression of GAPDH in Capan-2 cells by PTEN overexpression plasmid. The three samples on the left are the control group, and the three samples on the right are the PTEN overexpression plasmid transfection groups. (blots in Figure 5A)
